# Supplementary material for: Genomic ascertainment of PALB2-related cancer predisposition: PALB2-related cancer predisposition
Source: medRxiv. 2026 Apr 4:2026.04.03.26349984. Preprint. [Version 1] doi: 10.64898/2026.04.03.26349984 (PMC13060390; doi:10.64898/2026.04.03.26349984)
Supplement: Supplement 3 — Supplemental Figure 1. Power as a function of risk (odds ratio) in MyCode for a range of cancer rates. Prevalence data from cohort-specific frequency of PALB2-heterozygotes (Table 1). Dark gray line represents 80% power, and light gray line represents 90% power. [file media-3.pdf]

A.

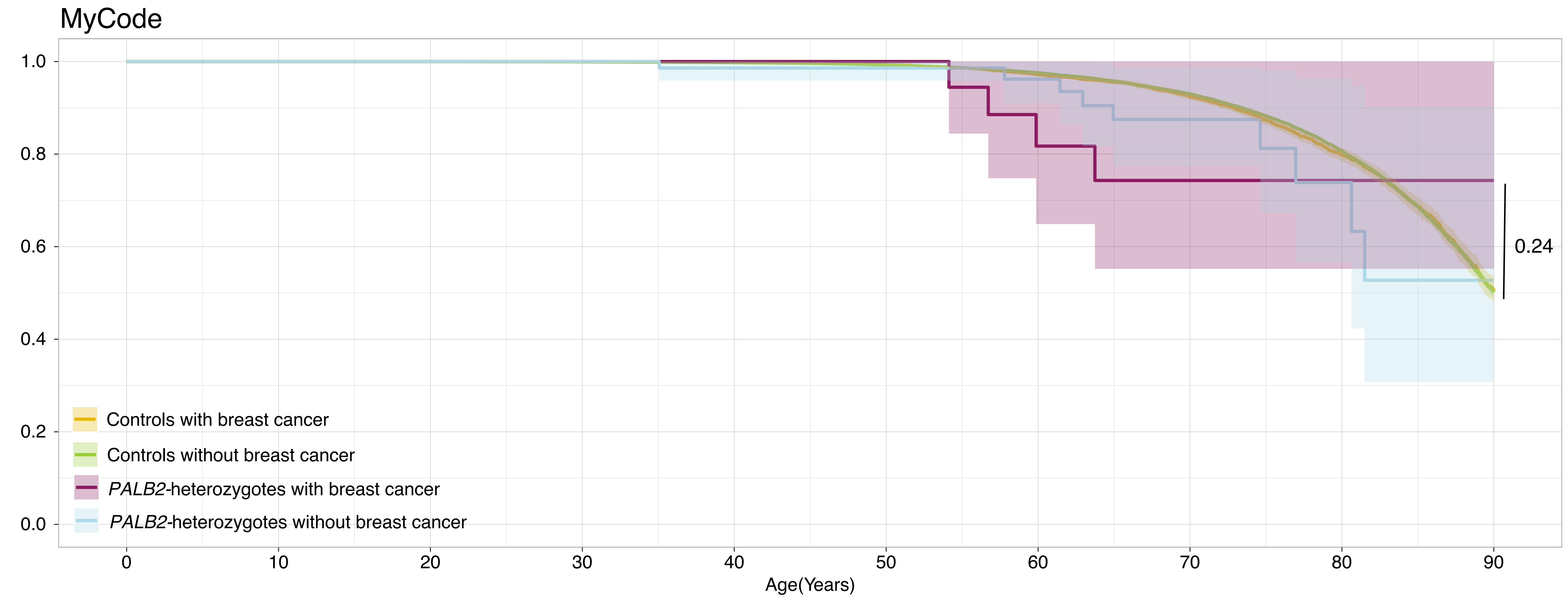

Number at risk (number censored)

	0	10	20	30	40	50	60	70	80	90
Controls with breast cancer	6146 (0)	6146 (0)	6146 (0)	6141 (3)	6087 (50)	5833 (271)	5024 (966)	3530 (2248)	1641 (3783)	394 (5049)
Controls without breast cancer	95136 (0)	95136 (0)	94948 (188)	90198 (4885)	76489 (18448)	62190 (32430)	45886 (47807)	27243 (64758)	11204 (78341)	2695 (86902)
PALB2-heterozygotes with breast cancer	23 (0)	23 (0)	23 (0)	23 (0)	22 (1)	20 (3)	12 (8)	6 (13)	3 (16)	1 (19)
PALB2-heterozygotes without breast cancer	89 (0)	89 (0)	89 (0)	80 (9)	63 (25)	46 (42)	39 (48)	20 (64)	8 (74)	1 (80)

B.

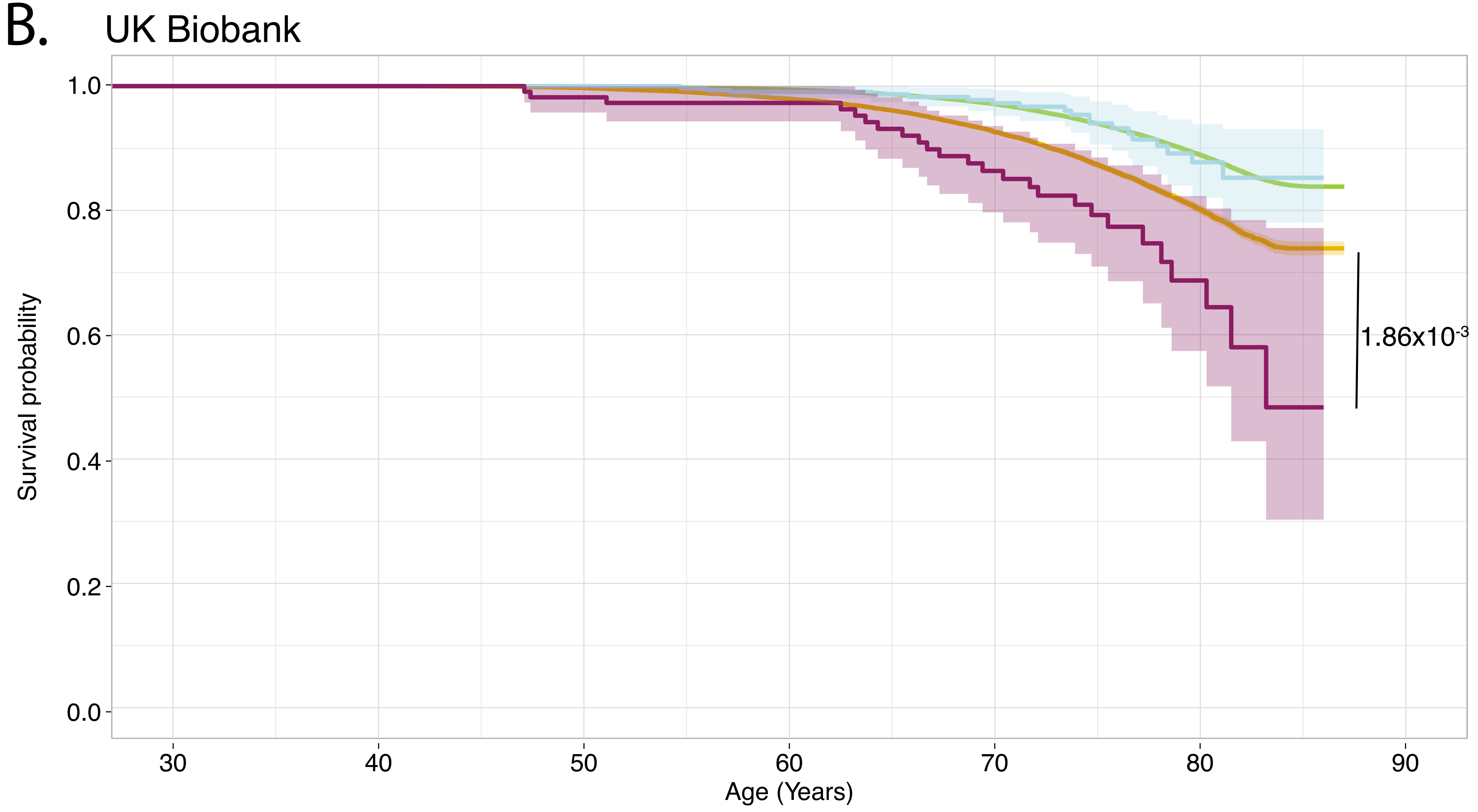

Number at risk (number censored)

	30	40	50	60	70	80	90
Controls with breast cancer	20020 (0)	20020 (0)	19958 (3)	18820 (1077)	14078 (5172)	4821 (13625)	0 (17213)
Controls without breast cancer	233996 (0)	233996 (0)	233721 (151)	213482 (25033)	143172 (93291)	44464 (186266)	0 (219992)
PALB2-heterozygotes with breast cancer	111 (0)	111 (0)	109 (0)	103 (9)	71 (31)	19 (73)	0 (86)
PALB2-heterozygotes without breast cancer	326 (0)	326 (0)	326 (0)	289 (42)	188 (138)	62 (265)	0 (307)
