# Supplementary figures and images for: Genomic ascertainment of PALB2-related cancer predisposition: PALB2-related cancer predisposition
Source: medRxiv. 2026 Apr 4:2026.04.03.26349984. Preprint. [Version 1] doi: 10.64898/2026.04.03.26349984 (PMC13060390; doi:10.64898/2026.04.03.26349984)

# UKBB

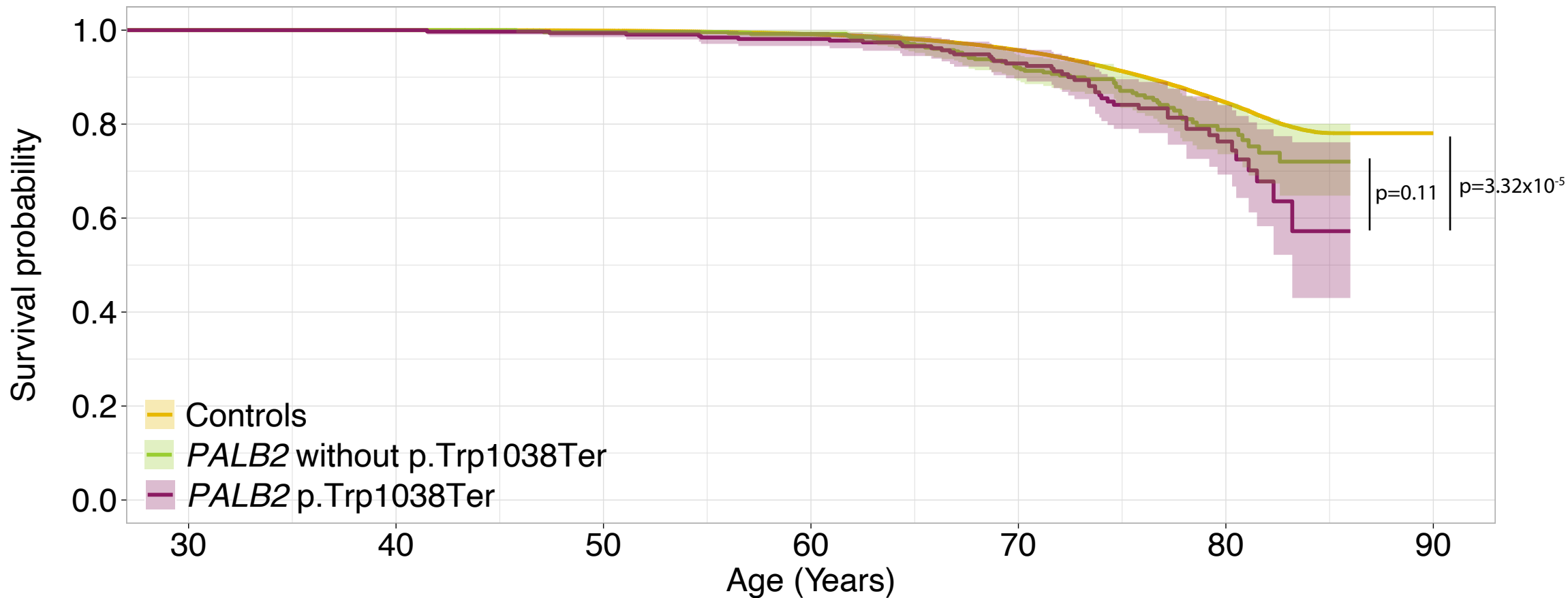

Number at risk (number censored)

|                                       |            |            |              |                |                 |                |            |
|---------------------------------------|------------|------------|--------------|----------------|-----------------|----------------|------------|
| Controls                              | 468658 (0) | 468658 (0) | 467966 (280) | 427249 (48754) | 290665 (176665) | 92871 (357994) | 1 (427575) |
| <i>PALB2</i> het without p.Trp1038Ter | 502 (0)    | 502 (0)    | 500 (0)      | 457 (54)       | 300 (185)       | 94 (372)       | 0 (439)    |
| <i>PALB2</i> p.Trp1038Ter             | 319 (0)    | 319 (0)    | 317 (0)      | 289 (31)       | 181 (126)       | 57 (240)       | 0 (274)    |

Supplement: Supplement 4 — Supplemental Figure 2. Power as a function of risk (odds ratio) in UK Biobank for a range of cancer rates. Prevalence data from cohort-specific frequency of PALB2-heterozygotes (Table 1). Dark gray line represents 80% power, and light gray line represents 90% power. [file media-4.pdf]

- All *PALB2*-heterozygotes (n=822)
- *PALB2*-heterozygotes without p.Trp1038Ter (n=503)
- *PALB2* p.Trp1038Ter (n=319)

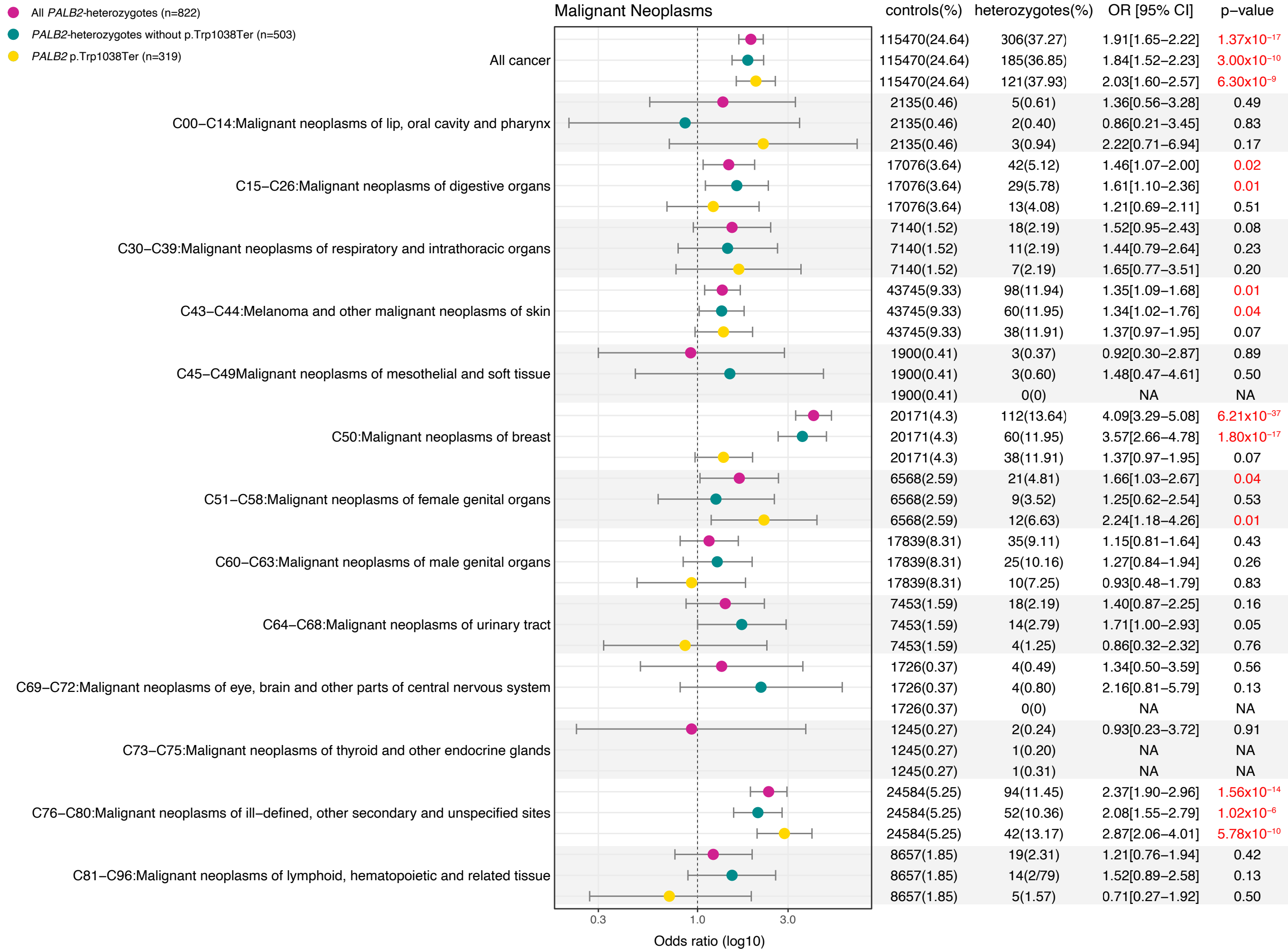

Supplement: Supplement 6 — Supplemental Figure 4. Power as a function of risk (odds ratio) in UK Biobank for a range of cancer rates. Prevalence data from cohort-specific frequency of PALB2-heterozygotes without PALB2 p.Trp1038Ter. Dark gray line represents 80% power, and light gray line represents 90% power. [file media-6.pdf]

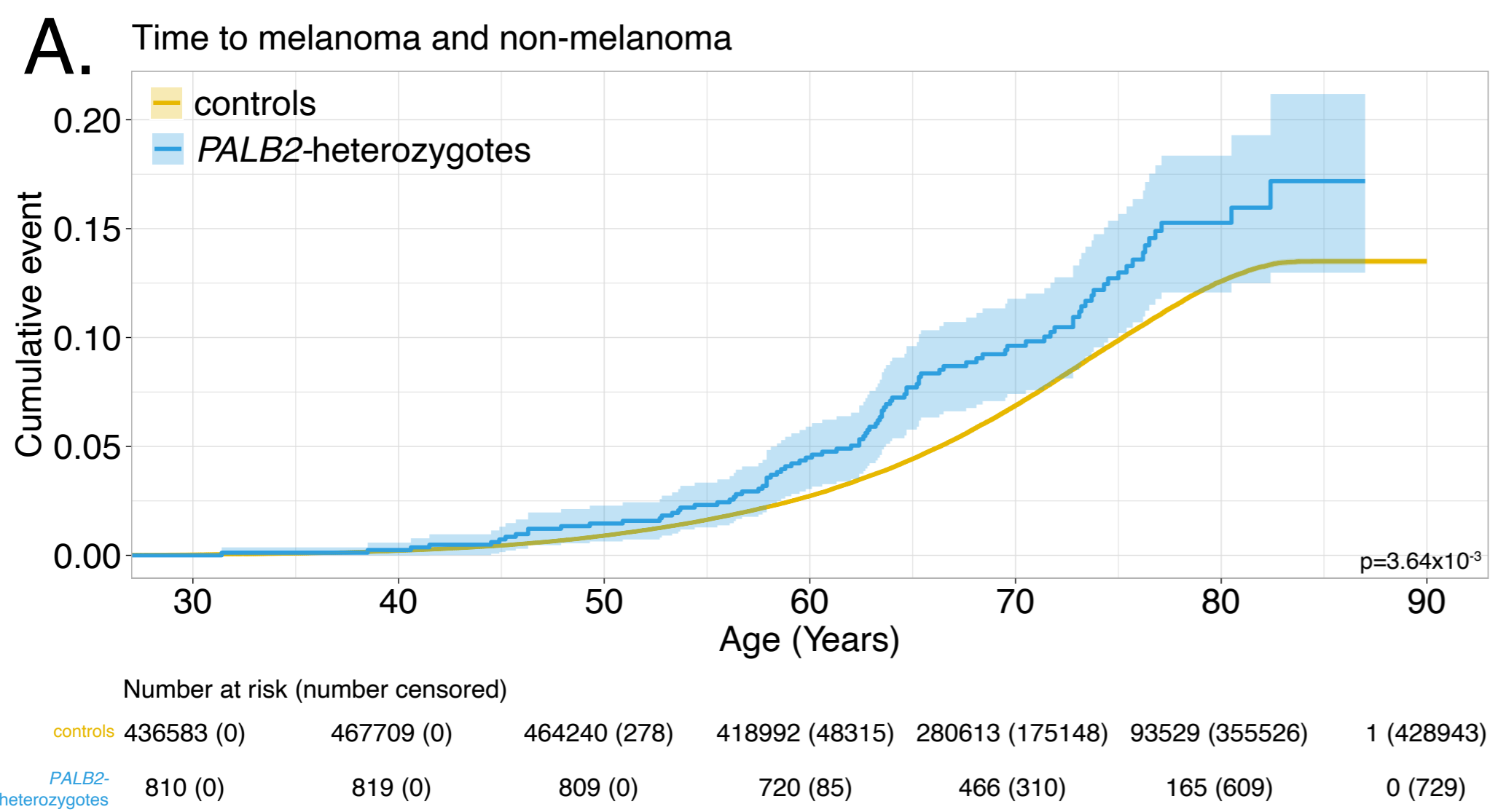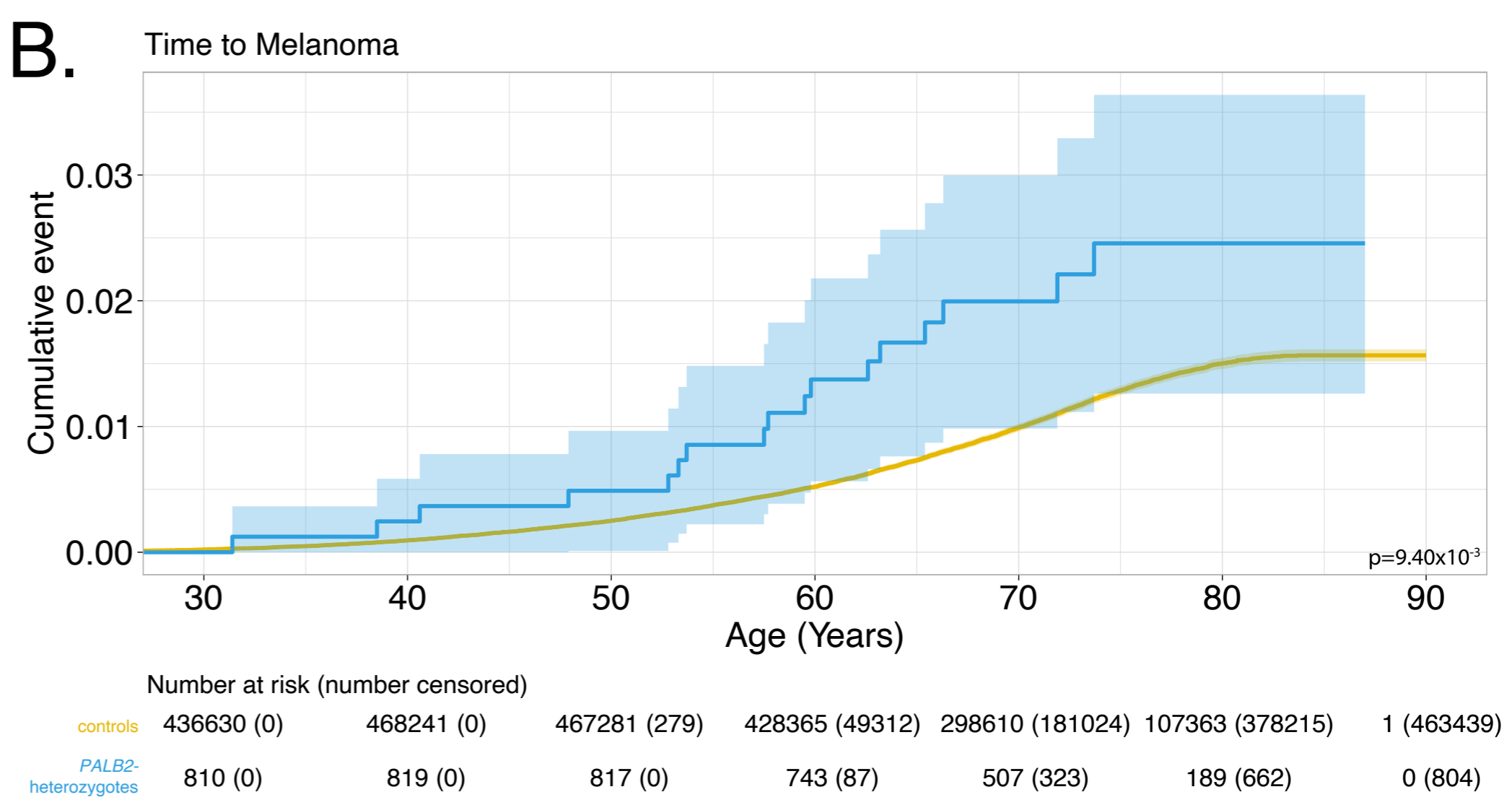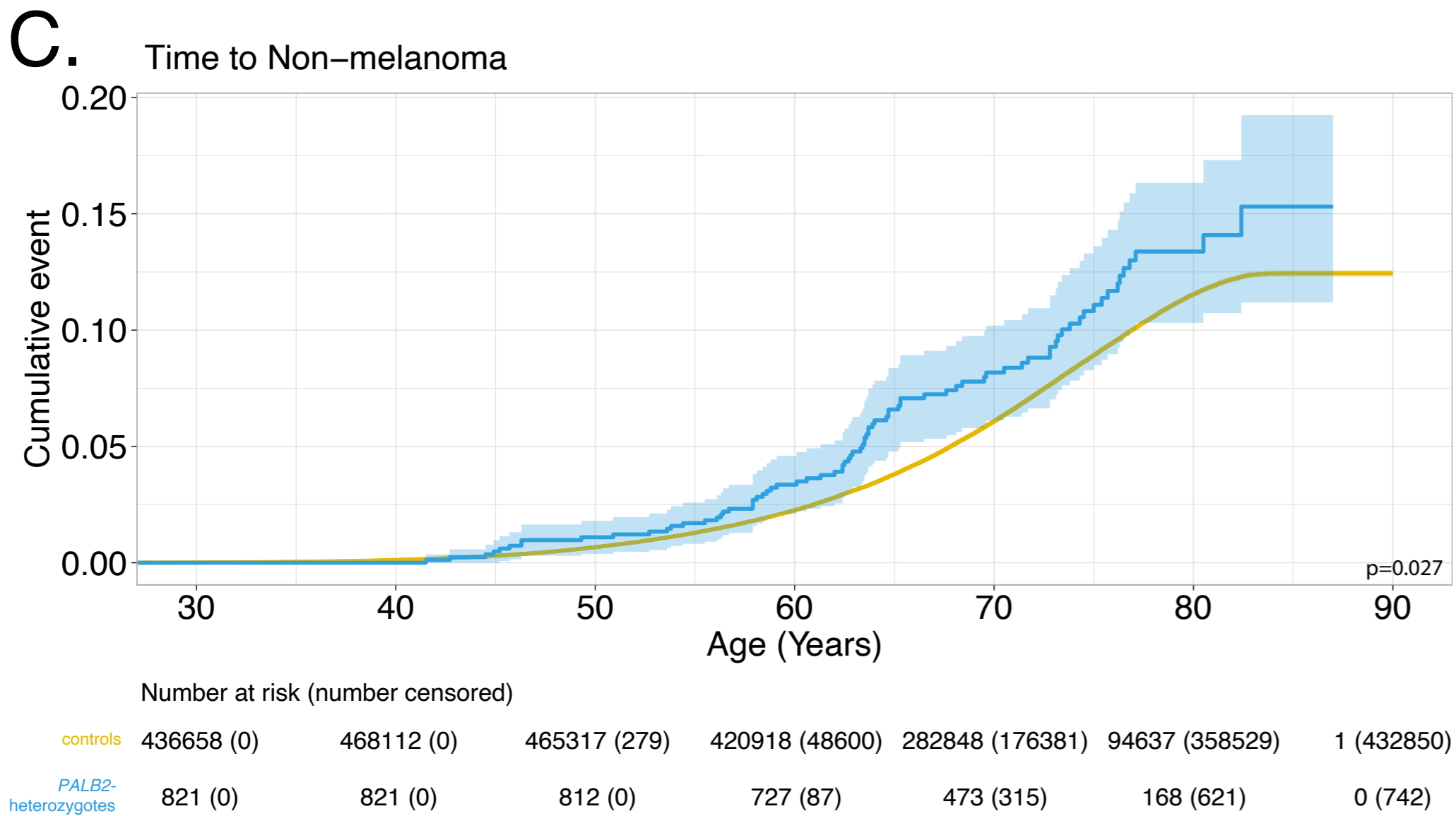

Supplement: Supplement 7 — Supplemental Figure 5. Expansion of odds ratio for PALB2-heterozygotes for organ system groupings of cancer ICD codes with a significant excess of risk in MyCode and UK Biobank. CI: 95% confidence interval; OR: odds ratio. All organs are listed for each organ system, even if no tumors were reported (e.g., C58: malignant neoplasm of placenta) [file media-7.pdf]

# Time to Pancreatic Cancer

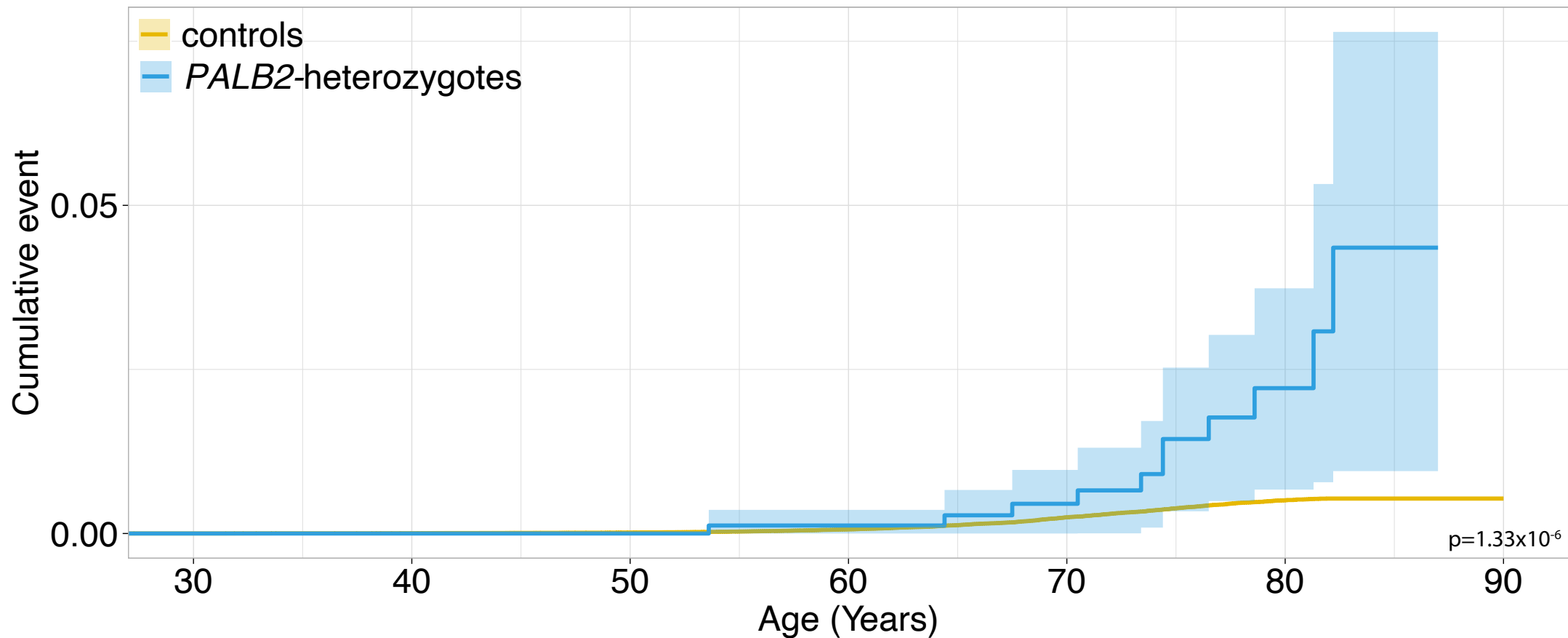

Number at risk (number censored)

|                             |            |            |              |                |                 |                 |            |
|-----------------------------|------------|------------|--------------|----------------|-----------------|-----------------|------------|
| controls                    | 463034 (0) | 468638 (0) | 468354 (280) | 430254 (49593) | 300692 (182215) | 108409 (381084) | 1 (467165) |
| <i>PALB2</i> -heterozygotes | 821 (0)    | 821 (0)    | 821 (0)      | 750 (90)       | 513 (330)       | 188 (671)       | 0 (810)    |

Supplement: Supplement 8 — Supplemental Figure 6. Odds ratio of PALB2-heterozygotes for carcinoma in situ codes in MyCode and UK Biobank [file media-8.pdf]

# PALB2 Benign Neoplasms

controls(%) heterozygotes(%) OR [95% CI] p-value

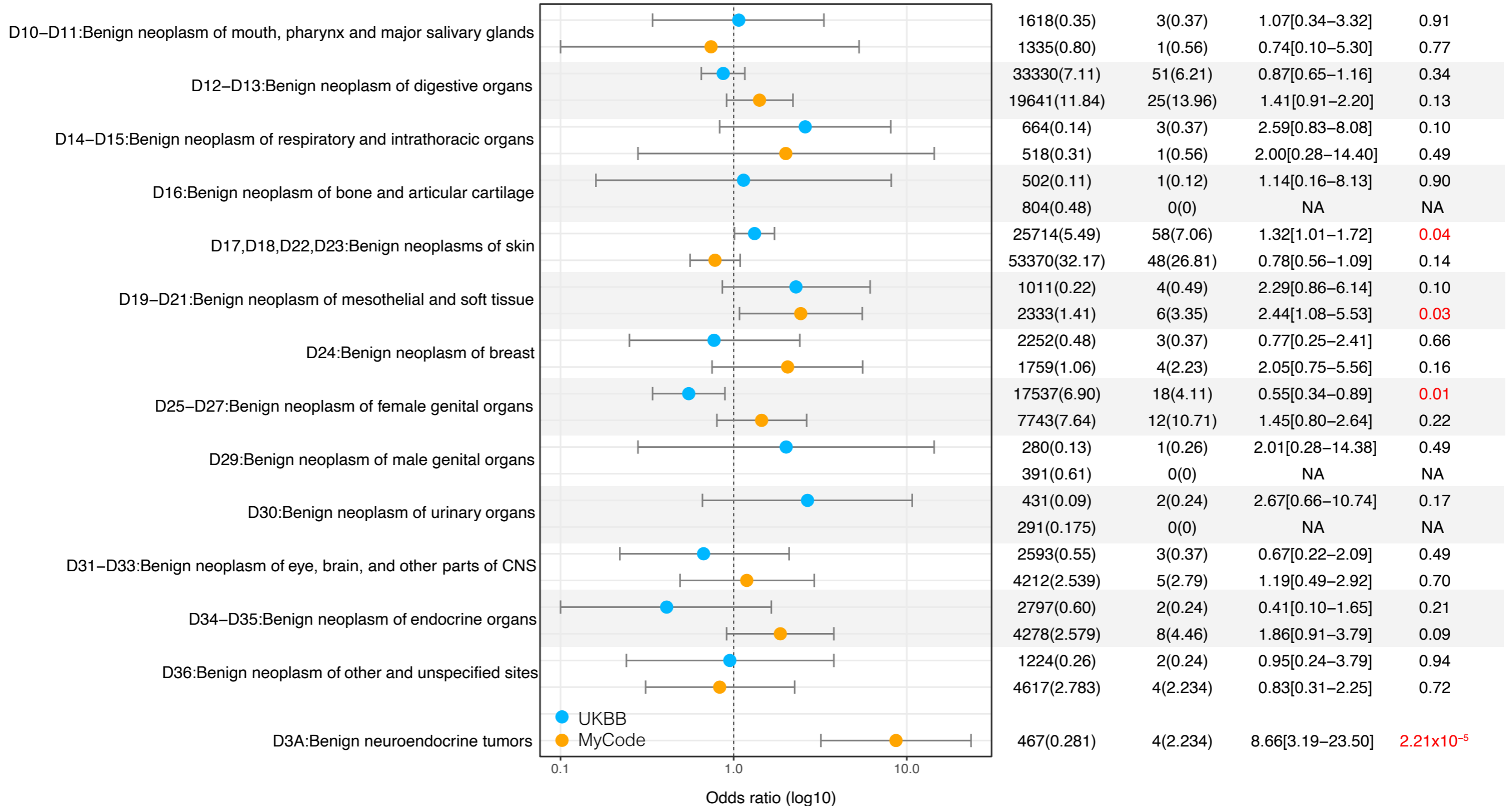

Supplement: Supplement 10 — Supplemental Figure 8. Odds ratio of PALB2-heterozygotes for tumors of uncertain behavior in MyCode and UK Biobank [file media-10.pdf]

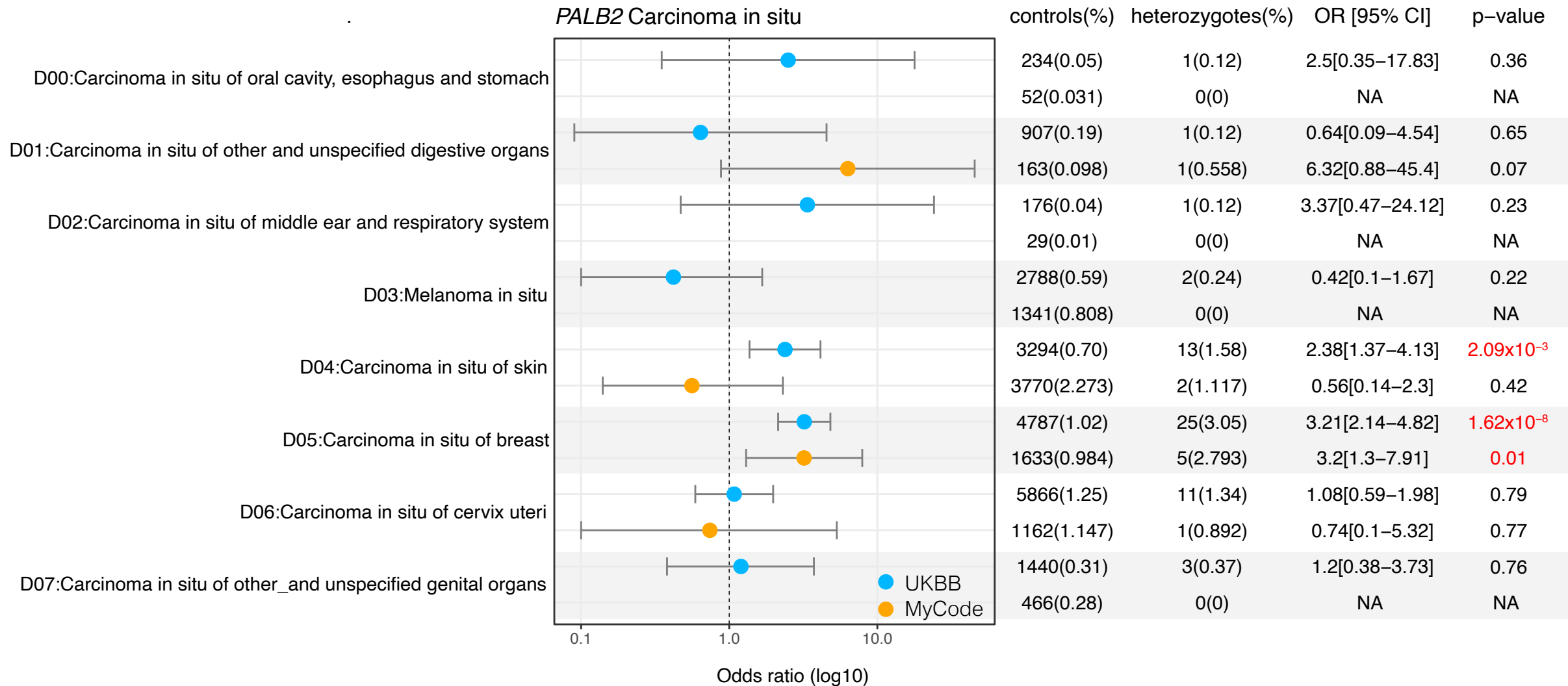

Supplement: Supplement 11 — Supplemental Figure 9. Time-to-pancreatic-cancer in PALB2-heterozygotes in the UK Biobank. See also Supplemental Table 7 [file media-11.pdf]

# UKBB *PALB2* without p.Trp1038Ter

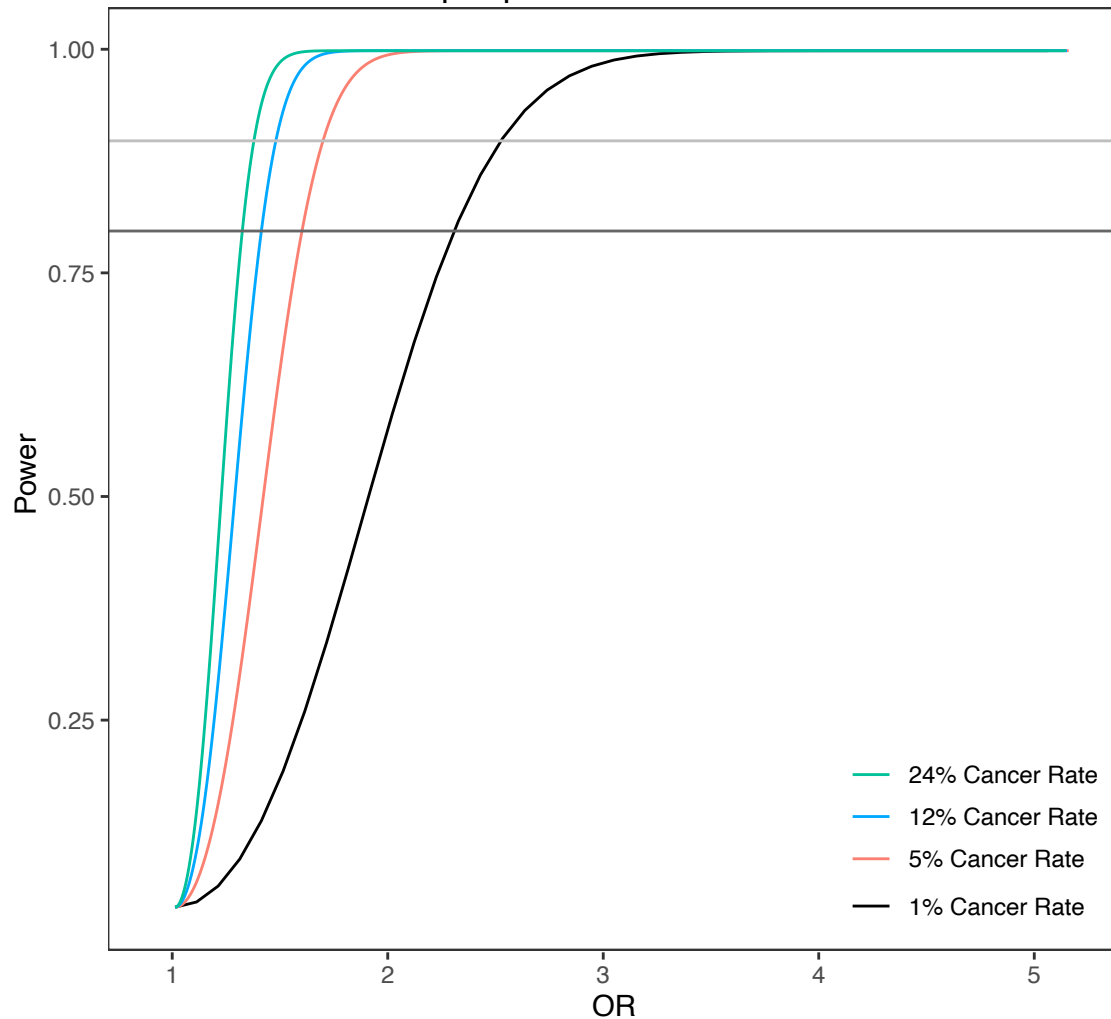

Supplement: Supplement 13 [file media-13.pdf]

# UKBB *PALB2* p.Trp1038Ter

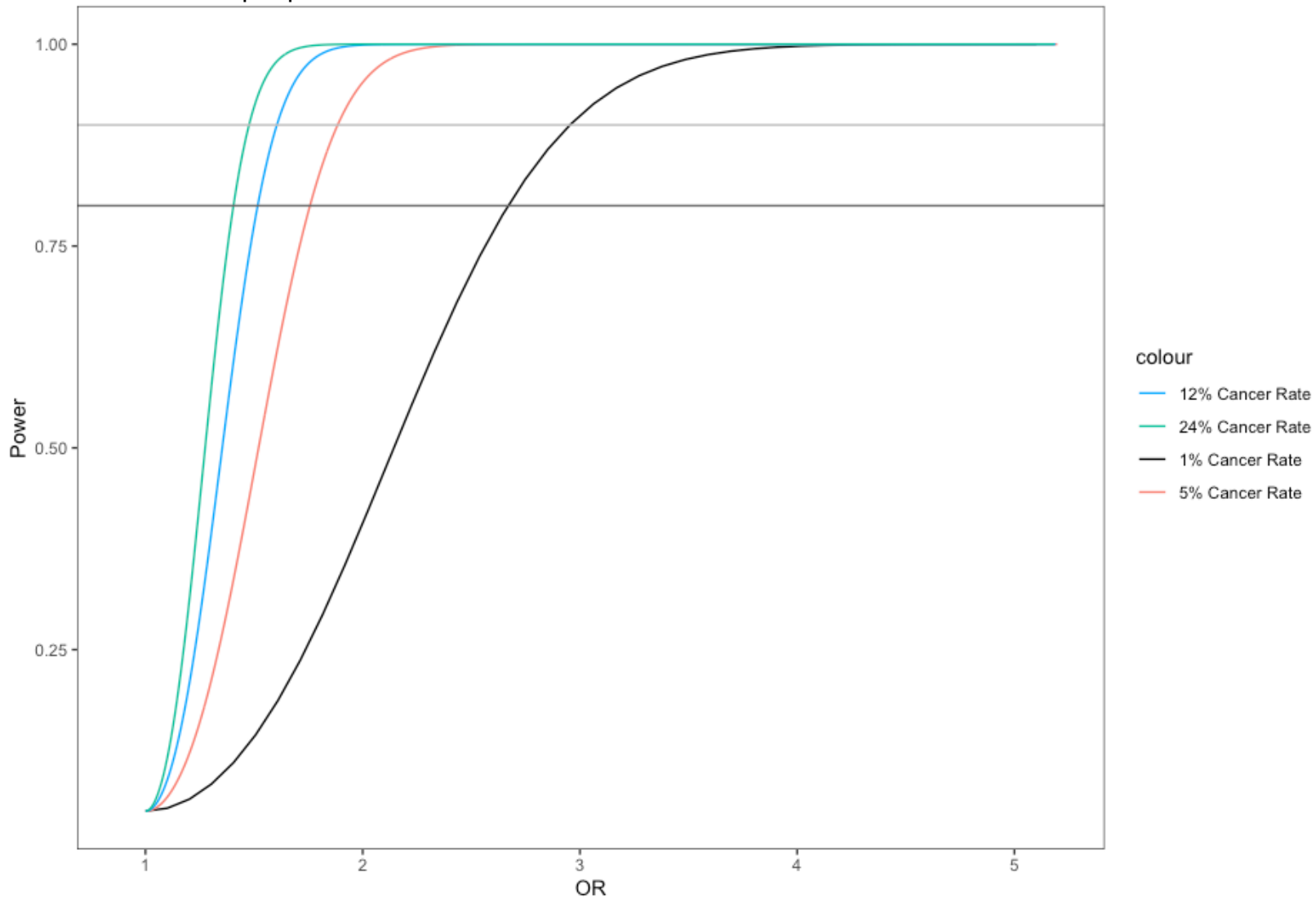

Supplement: Supplement 14 — Supplemental Figure 12. Time-to-cancer for All PALB2-heterozygotes without PALB2 Trp1038Ter and PALB2 Trp1038Ter heterozygotes vs controls (panel A) and time-to-breast cancer (female only) for All PALB2-heterozygotes without PALB2 Trp1038Ter and PALB2 Trp1038Ter heterozygotes vs controls (panel B). See also Supplemental Table 9 and Supplemental Table 10. [file media-14.pdf]

# UKBB PALB2

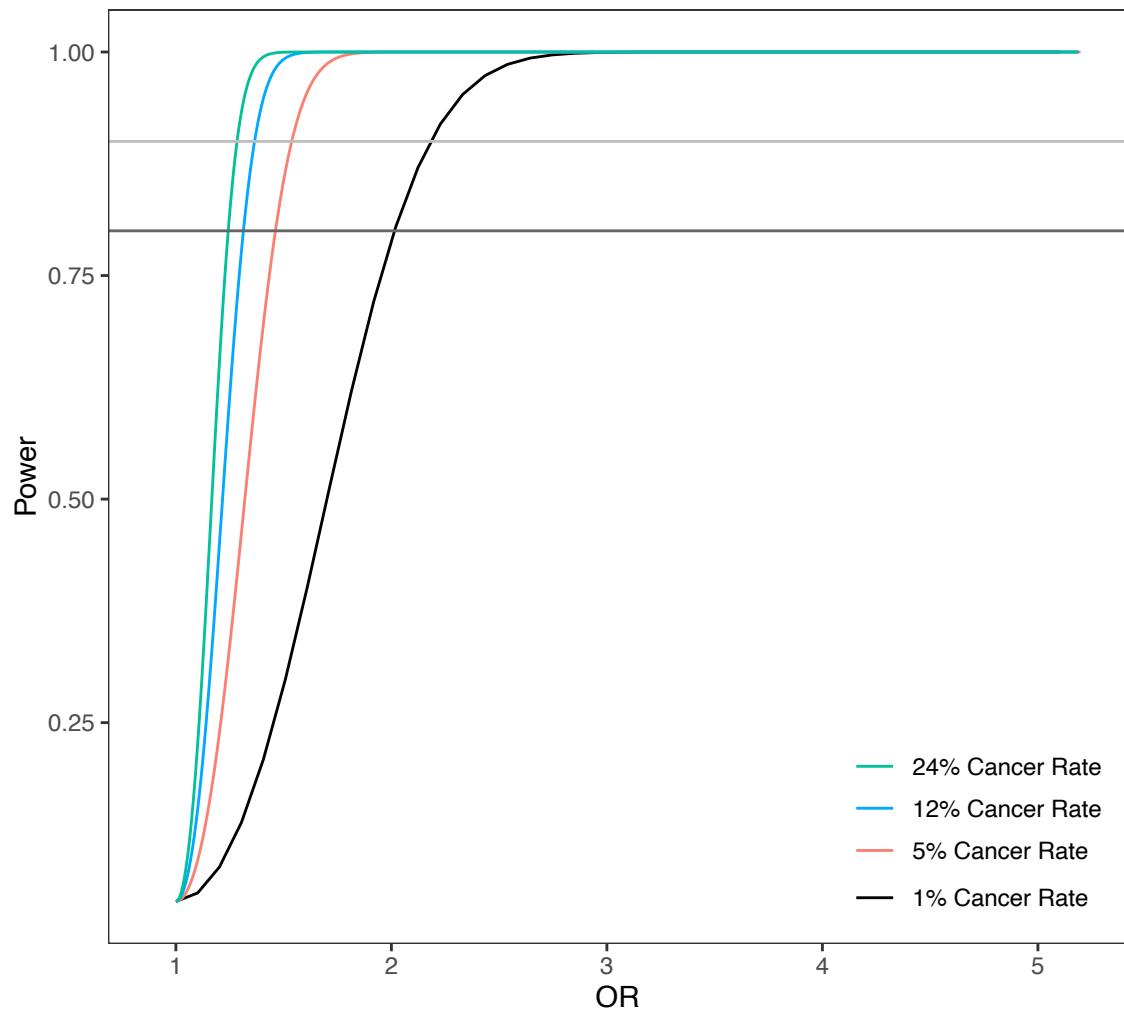

Supplement: Supplement 15 — Supplemental Figure 13. All-cause mortality in UK Biobank for All PALB2-heterozygotes without PALB2 Trp1038Ter and PALB2 Trp1038Ter heterozygotes vs controls. See also Figure 5 (all-cause mortality for PALB2-heterozygotes). [file media-15.pdf]

# MyCode

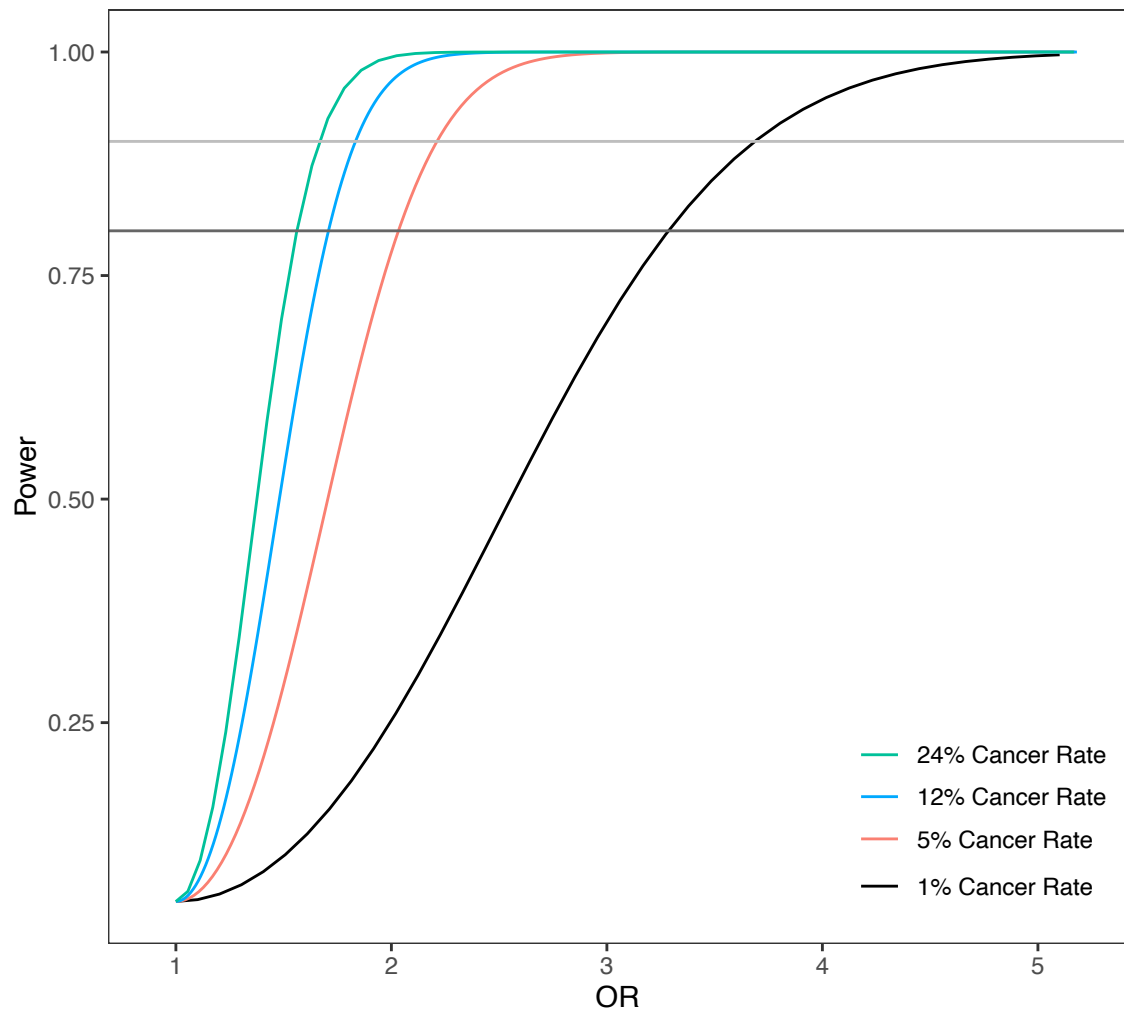

Supplement: Supplement 16 — Supplemental Figure 14. All-cause mortality in PALB2-heterozygotes with and without breast cancer vs controls with and without breast cancer in MyCode (panel A) and UK Biobank (panel B). [file media-16.pdf]
