# Supplementary material for: Genomic ascertainment of PALB2-related cancer predisposition: PALB2-related cancer predisposition
Source: medRxiv. 2026 Apr 4:2026.04.03.26349984. Preprint. [Version 1] doi: 10.64898/2026.04.03.26349984 (PMC13060390; doi:10.64898/2026.04.03.26349984)
Supplement: Supplement 5 — Supplemental Figure 3. Power as a function of risk (odds ratio) in UK Biobank for a range of cancer rates. Prevalence data from cohort-specific frequency of PALB2 p.Trp1038Ter. Dark gray line represents 80% power, and light gray line represents 90% power. [file media-5.pdf]

A.

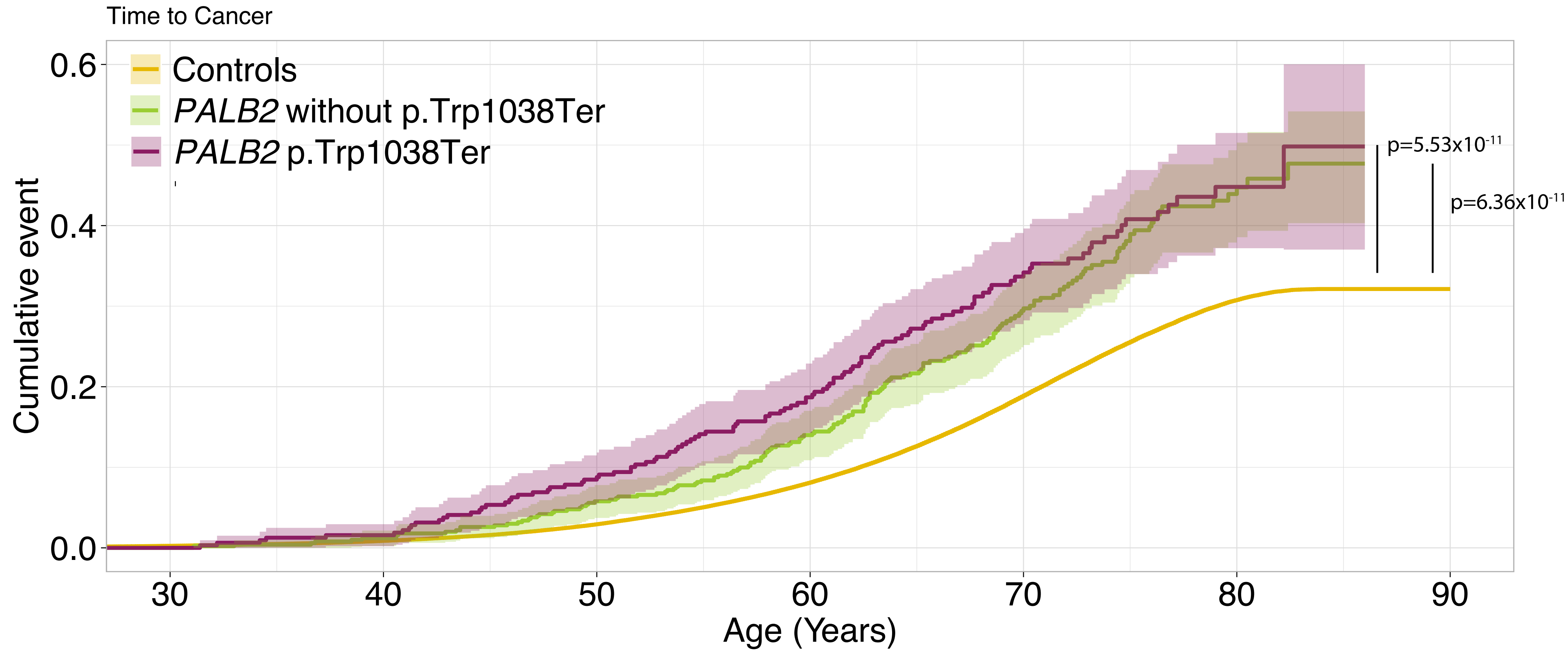

Number at risk (number censored)

Controls	435908 (0)	465000 (0)	455205 (272)	396404 (45917)	244672 (161520)	72119 (311554)	1 (367391)
PALB2 het without p.Trp1038Ter	498 (0)	496 (0)	473 (0)	397 (47)	227 (156)	68 (287)	0 (336)
PALB2 p.Trp1038Ter	312 (0)	314 (0)	292 (0)	241 (24)	128 (102)	39 (181)	0 (207)

B.

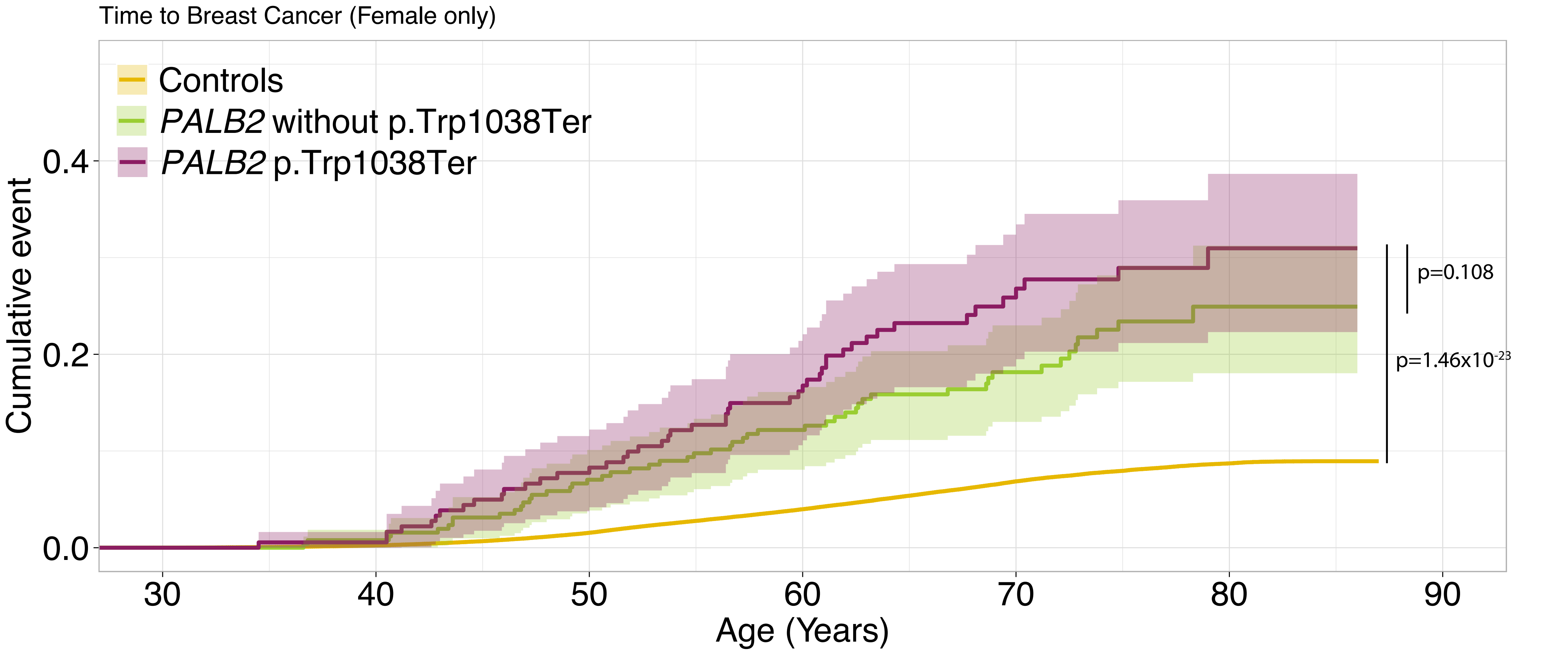

Number at risk (number censored)

Controls	237877 (0)	253419 (0)	250008 (151)	224488 (25509)	150427 (96124)	50413 (196364)	0 (236187)
PALB2 het without p.Trp1038Ter	256 (0)	254 (0)	238 (0)	204 (27)	135 (86)	43 (173)	0 (205)
PALB2 p.Trp1038Ter	181 (0)	180 (0)	167 (0)	139 (15)	80 (60)	29 (116)	0 (134)
