# Supplementary material for: Genomic ascertainment of PALB2-related cancer predisposition: PALB2-related cancer predisposition
Source: medRxiv. 2026 Apr 4:2026.04.03.26349984. Preprint. [Version 1] doi: 10.64898/2026.04.03.26349984 (PMC13060390; doi:10.64898/2026.04.03.26349984)
Supplement: Supplement 12 — Supplemental Figure 10. Time-to-skin-cancer (panel A), time-to-melanoma (panel B) and time-to-non- melanoma (Panel C) in PALB2-heterozygotes in the UK Biobank. See also Supplemental Table 8. [file media-12.pdf]

. *PALB2* Malignant Neoplasms

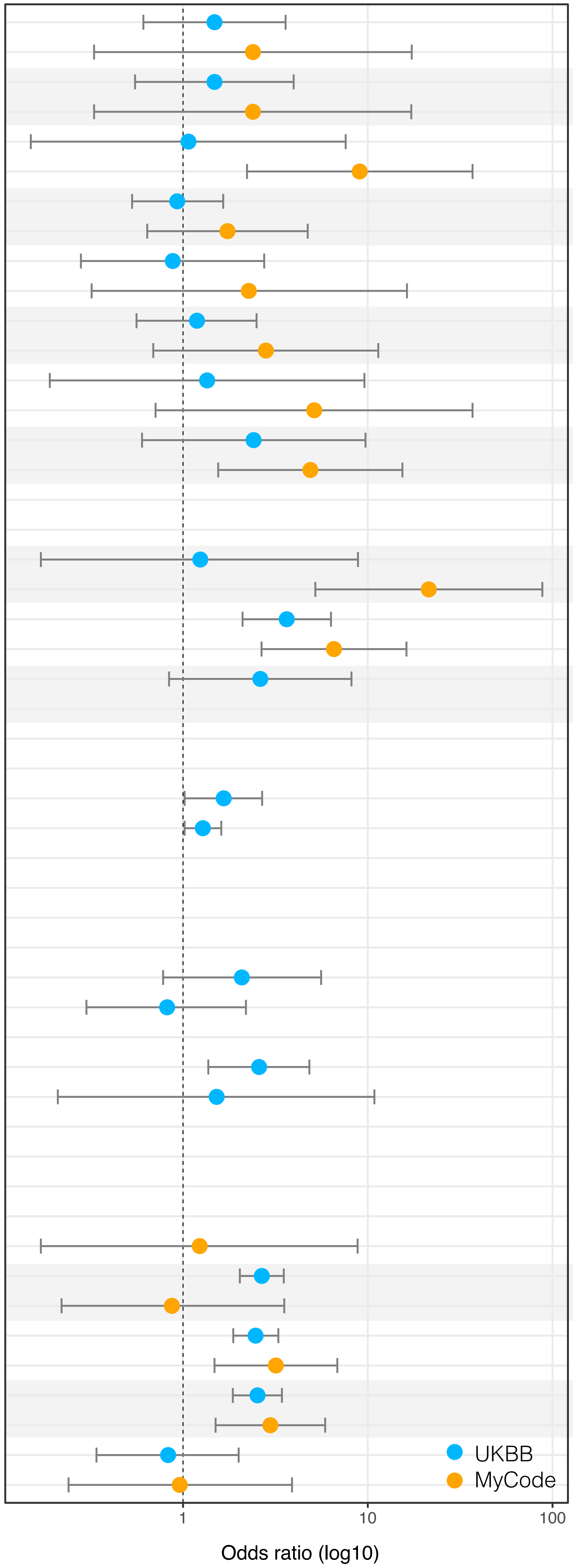

	controls(%)	heterozygotes(%)	OR [95% CI]	p–value
C15:Malignant neoplasm of esophagus	1944(0.41)	5(0.61)	1.48[0.61–3.59]	0.38
	446(0.268)	1(0.56)	2.39[0.33–17.30]	0.39
C16:Malignant neoplasm of stomach	1558(0.33)	4(0.49)	1.48[0.55–3.97]	0.44
	441(0.27)	1(0.56)	2.39[0.33–17.20]	0.39
C17:Malignant neoplasm of small intestine	543(0.12)	1(0.12)	1.07[0.15–7.59]	0.95
	229(0.14)	2(1.12)	9.04[2.22–36.90]	2.13×10^{-3}
C18:Malignant neoplasm of colon	7490(1.60)	12(1.46)	0.93[0.53–1.65]	0.81
	2488(1.5)	4(2.23)	1.74[0.64–4.73]	0.28
C19:Malignant neoplasm of rectosigmoid junction	1970(0.42)	3(0.37)	0.88[0.28–2.75]	0.83
	455(0.274)	1(0.56)	2.27[0.32–16.30]	0.41
C20:Malignant neoplasm of rectum	3429(0.73)	7(0.85)	1.19[0.56–2.50]	0.65
	742(0.447)	2(1.12)	2.81[0.69–11.40]	0.15
C21:Malignant neoplasm of anus and anal canal	436(0.09)	1(0.12)	1.35[0.19–9.59]	0.77
	203(0.12)	1(0.56)	5.13[0.71–36.90]	0.1
C22:Malignant neoplasm of liver and intrahepatic bile ducts	497(0.20)	2(0.46)	2.41[0.60–9.72]	0.22
	652(0.393)	3(1.68)	4.89[1.55–15.40]	0.01
C23:Malignant neoplasm of gallbladder	67(0.03)	0(0)	NA.	NA.
	46(0.03)	0(0)	NA.	NA.
C24:Malignant neoplasm of other and unspecified parts_of biliary tract	468(0.10)	1(0.12)	1.24[0.17–8.85]	0.83
	101(0.06)	2(1.12)	21.40[5.20–88.10]	2.22×10^{-5}
C25:Malignant neoplasm of pancreas	2113(0.45)	13(1.58)	3.64[2.10–6.32]	4.38×10^{-6}
	827(0.498)	5(2.79)	6.56[2.66–16.2]	4.33×10^{-5}
C26:Malignant neoplasm of other and ill–defined digestive organs	668(0.14)	3(0.37)	2.62[0.84–8.16]	0.1
	60(0.036)	0(0)	NA.	NA.
C43:Melanoma	6004(1.28)	17(2.07)	1.66[1.02–2.68]	0.04
C44:Other malignant neoplasms of skin	39738(8.48)	85(10.35)	1.28[1.02–1.61]	0.04
C51:Malignant neoplasm of vulva	292(0.11)	0(0)	NA.	NA.
C52:Malignant neoplasm of vagina	78(0.03)	1(0.22)	NA.	0.97
C53:Malignant neoplasm of cervix uteri	1143(0.45)	4(0.92)	2.08[0.78–5.59]	0.14
C54:Malignant neoplasm of corpus uteri	2894(1.14)	5(1.14)	0.82[0.3–2.19]	0.69
C55:Malignant neoplasm of uterus, part unspecified	335(0.13)	0(0)	NA.	NA.
C56:Malignant neoplasm of ovary	2333(0.92)	12(2.75)	2.58[1.37–4.83]	3.19×10^{-3}
C57:Malignant neoplasm of other and unspecified female genetial organs	392(0.15)	1(0.23)	1.52[0.21–10.86]	0.68
C58:Malignant neoplasm of placenta	25(0.01)	0(0)	NA.	NA.
C76:Malignant neoplasm of other and ill–defined sites	319(0.07)	0(0)	NA.	NA.
	829(0.499)	1(0.558)	1.23[0.17–8.81]	0.84
C77:Secondary and unspecified malignant neoplasm of lymph nodes	12837(2.74)	57(6.94)	2.67[2.03–3.51]	1.84×10^{-12}
	2305(1.389)	2(1.117)	0.87[0.22–3.53]	0.85
C78:Secondary malignant neoplasm of respiratory and digestive_organs	13127(2.80)	54(6.58)	2.47[1.87–3.28]	2.86×10^{-10}
	2362(1.424)	7(3.91)	3.18[1.48–6.84]	3.00×10^{-3}
C79:Secondary malignant neoplasm of other and unspecified sites	10596(2.26)	44(5.36)	2.53[1.86–3.43]	3.35×10^{-9}
	3340(2.013)	9(5.027)	2.97[1.5–5.88]	1.81×10^{-3}
C80:Malignant neoplasm without specification of site	3525(0.75)	5(0.61)	0.83[0.34–2.00]	0.67
	2148(1.29)	2(1.117)	0.96[0.24–3.89]	0.96
